# Comparative analysis of gut DNA viromes in wild and captive Himalayan vultures

**DOI:** 10.3389/fmicb.2023.1120838

**Published:** 2023-08-02

**Authors:** Jundie Zhai, You Wang, Boyu Tang, Sisi Zheng, Shunfu He, Wenxin Zhao, Hanxi Chen, Jun Lin, Feng Li, Yuzi Bao, Zhuoma Lancuo, Kirill Sharshov, Chuanfa Liu, Wen Wang

**Affiliations:** ^1^State Key Laboratory of Plateau Ecology and Agriculture, Qinghai University, Xining, Qinghai, China; ^2^College of Eco-Environmental Engineering, Qinghai University, Xining, Qinghai, China; ^3^Animal Disease Prevention and Control Center of Qinghai Province, Xining, Qinghai, China; ^4^Xining Wildlife Park of Qinghai Province, Xining, Qinghai, China; ^5^College of Finance and Economics, Qinghai University, Xining, Qinghai, China; ^6^Federal Research Center of Fundamental and Translational Medicine, Novosibirsk, Russia; ^7^College of Life Sciences, University of Chinese Academy of Sciences, Beijing, China

**Keywords:** *Gyps himalayensis*, viral metagenomics, phage, zoo, scavenger, conservation biology, high-throughput sequencing technology

## Abstract

**Introduction:**

Himalayan vultures (*Gyps hinalayensis*) are widely distributed on the Qinghai-Tibetan Plateau and play a crucial role in maintaining the ecological balance by feeding on decayed corpses of wild and domestic animals. Large-scale culture and metagenomics studies have broadened our understanding of viral diversity in animals’ gastrointestinal tracts. However, despite the importance of gut viral communities in regulating bacterial diversity and performing symbiotic functions, no gut viral study has been conducted on Himalayan vultures. Furthermore, the impact of captivity on the gut virome of these vultures remains unknown.

**Methods:**

In this study, metagenomic sequencing methods targeting DNA of virus-like particles enriched from feces were used to characterize the gut DNA viromes of wild and captive Himalayan vultures.

**Results:**

In total, 22,938 unique viral operational taxonomic units (vOTUs) were identified and assigned to 140 viral genera in 41 viral families. These families included viruses associated with bacteria, animals, plants, insects, and archaea. Phage communities, including *Siphoviridae*, *Microviridae*, *Myoviridae*, *Inoviridae*, and *Herelleviridae*, dominated the gut virome of Himalayan vultures. Wild vultures exhibited higher viral richness and diversity compared with those in captivity. The functional capacity of the gut virome was characterized by identifying 93 KEGG pathways, which were significantly enriched in metabolism and genetic information processing. Abundant auxiliary metabolic genes, such as carbohydrate-active enzyme, and antibiotic resistance genes, were also found in the vultures’ gut virome.

**Discussion:**

Our findings reveal the complex and diverse viral community present in the gut virome of Himalayan vultures, which varies between wild, and captive states. The DNA virome dataset establishes a baseline for the vultures’ gut virome and will serve as a reference for future virus isolation and cultivation. Understanding the impact of captivity on the gut virome contributes to our knowledge of vultures’ response to captivity and aids in optimizing their rehabilitation and implementing protective measures.

## 1. Introduction

Vultures, as nature’s most successful obligate scavenging birds, play crucial ecological, cultural, and public health roles by consuming rotting carcasses in natural and anthropogenic environments (García-Jiménez et al., 2022). With 23 vulture species worldwide, evolved from separate phylogenetic lineages including New World vultures (7 species in the family Cathartidae) and Old World vultures (16 species in the family Accipitridae) (Jarvis et al., 2014), these avian groups face significant threats. Specifically, approximately 73% of vulture species are at a high risk of extinction, with 77% of global populations experiencing dramatic declines in the late 20th century (Buechley and Sekercioglu, 2016). The decline is attributed to various factors such as dietary toxins [residual veterinary drugs in livestock carcasses (Swan et al., 2006; Adawaren et al., 2018) and poisonous baits (Margalida et al., 2019; van den Heever et al., 2019)], habitat loss, lack of food resources, nest abandonment, low reproductive rate, hunting for traditional medicine or belief-based use (Mckean et al., 2013), climate change (Marneweck et al., 2021), heavy metal bioaccumulation (Yamac et al., 2019; Bassi et al., 2021), and collisions with wind turbines (de Lucas et al., 2012). Consequently, political measures, such as banning the veterinary use of diclofenac in India, Pakistan, Nepal, and Bangladesh, as well as establishing supplementary feeding stations, are being implemented for vulture protection and conservation efforts (Cortes-Avizanda et al., 2010; Moleon et al., 2014; Morales-Reyes et al., 2018; Blanco et al., 2019).

Eight Old World vulture species inhabit China, with three species (*Gypaetus barbatus*, *Aegypius monachus*, and *Gyps himalayensis*) widely distributed in the Qinghai-Tibetan Plateau and performing the crucial ecological function of removing the rotting carcasses of domestic animals (e.g., yaks and Tibetan sheep) and wildlife, which could be disease sources, from the plateau. Among these three vulture species, the Himalayan vulture *G. himalayensis* has larger populations compared with the other species (Lu et al., 2009) and is more accessible for scientific research. Currently listed as “Near Threatened” on the International Union for Conservation of Nature’s Red List, Himalayan vultures may reach “Vulnerable” status owing to ongoing population decline (Paudel et al., 2016). However, limited research on this species hampers effective conservation efforts.

The gastrointestinal tracts of animals harbor complex microbial ecosystems (viruses, bacteria, archaea, fungi, and protists), forming coevolutionary relationships with hosts and playing vital roles in digestion, nutrient acquisition, immune regulation, development, disease resistance, and overall health (Colston and Jackson, 2016; Kumar et al., 2022). Although research on gut bacterial microbiomes in wild birds (representing 30% of known tetrapod diversity) is progressing rapidly (Bodawatta et al., 2021), studies on gut virome diversity (viral metagenomes) in birds have progressed comparatively slowly. However, the importance of studying viromes in birds and other wild animals has increased owing to the need for better prevention and control of emerging viral infectious diseases originating from wildlife (Albery et al., 2021; Chaffringeon et al., 2021; Li et al., 2021). The emergence of next-generation sequencing and improved bioinformatics tools has facilitated the identification of eukaryotic and prokaryotic viral sequences, revealing new pathogenic viruses in various bird species as well as their role in viral disease transmission (Shan et al., 2022; Zhu et al., 2022). However, our understanding of bird viromes is still in its early stages (François and Pybus, 2020).

Previous studies have shown that scavenging vultures could be potential reservoirs of bacterial pathogens due to their feeding habits (Roggenbuck et al., 2014; Zepeda Mendoza et al., 2018; Wang et al., 2021). However, the gut viromes of vultures, as vital components of the microbiome, remain poorly understood. Therefore, understanding the viral composition, and function associated with vultures is essential for conservation efforts. The host–gut virome alliance is influenced by various factors, including host genetics, diet, geographical environment, age, gender, lifestyle, and physiological status (Perlman et al., 2022). Additionally, captivity has been shown to alter the gut microbial composition and diversity in various vertebrates, including mammals, birds, fish, reptiles, and amphibians, potentially due to differences in diets and living environments in captivity compared with the wild (Hird, 2017; McKenzie et al., 2017). Therefore, it is important to investigate the changes that occur in gut viromes between wild Himalayan vultures and those in captivity, where artificially provided food is relatively clean and fresh compared with the complex scavenging diets of wild populations. In this study, we aimed to analyze the composition, diversity, and function of the gut virome in Himalayan vultures in different living environments, comparing captive and wild populations. Using viral metagenomic sequencing, given its capacity for detecting known and unknown viruses in the animal host, we aimed to (1) investigate gut viral compositional and functional variations between captive and wild Himalayan vultures, and (2) analyze the phylogenetic diversity of the main virus groups. To the best of our knowledge, this is the first study to analyze and compare the gut virome of Himalayan vultures in different living environments; therefore, our findings will contribute to our understanding of the coevolution and transmission of the gut virome in scavenging vultures and improve management knowledge regarding gut viromes in enteric diseases of captive individuals.

## 2. Materials and methods

### 2.1. Ethics statement

This study adhered to the guidelines for the care and use of experimental animals established by the Ministry of Science and Technology of the People’s Republic of China (approval number: 2006-398). The research protocol received approval from the Ethical Committee of Qinghai University. No capture, direct manipulation, or disturbance of Himalayan vultures occurred during this study.

### 2.2. Samples collection

**Figure 1:**
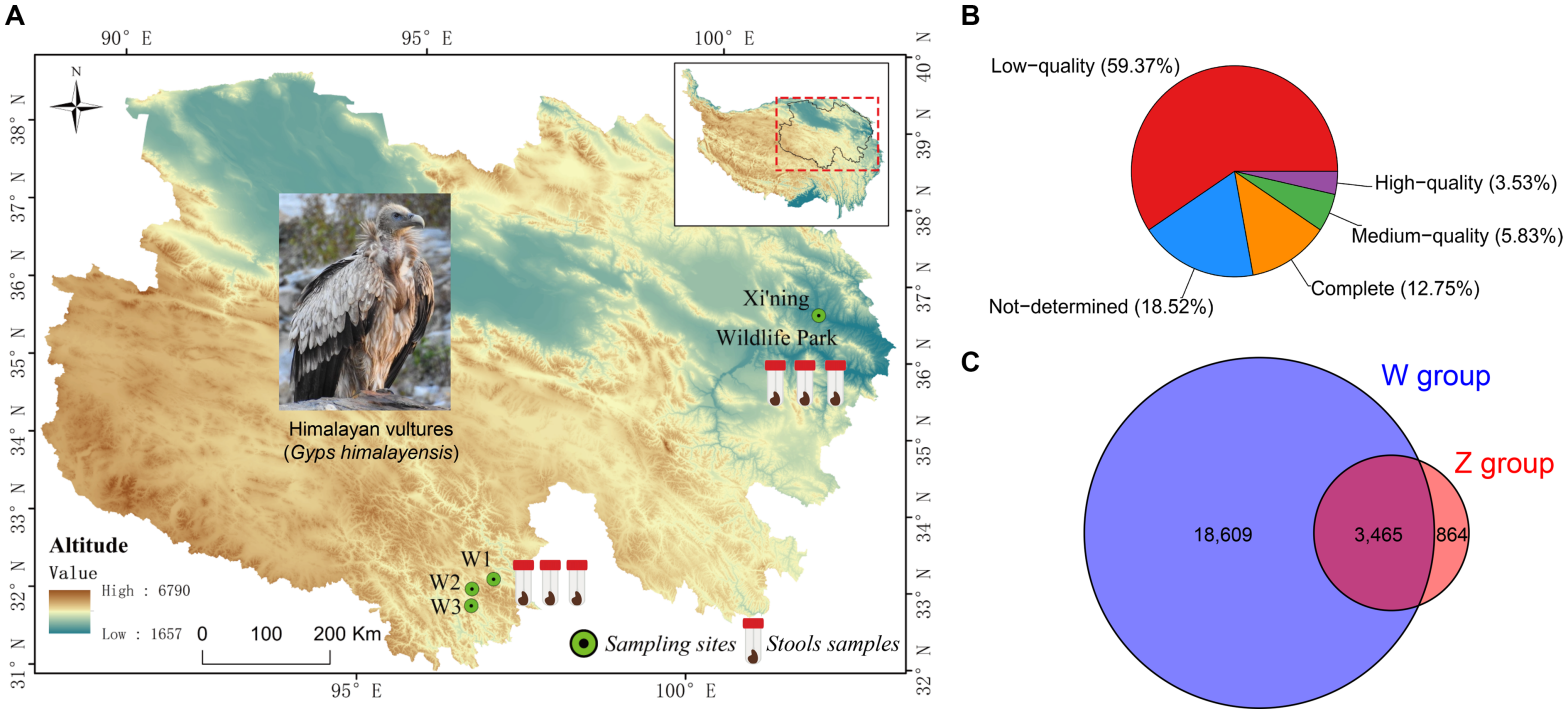
Map of the Qinghai-Tibetan Plateau with sampling sites **(A)**, and the results of the completeness of viral Operational Taxonomic Units (vOTUs) evaluated by CheckV **(B)**, and the Venn diagram of shared and unique vOTUs in the wild and zoo groups **(C)**.

In total, we collected 12 fresh fecal samples from six Himalayan vultures, comprised of three individuals from each captive and wild population (Figure 1A). Two fecal samples per individual were pooled to increase the total material available for DNA extraction. Feces were opportunistically collected from captive-reared Himalayan vultures after feeding housed at the Xi’ning Wildlife Park in China. No antibiotics or other medications were administered to the captive vultures for 6 months before the study. Feces from wild individuals were collected during a field survey of Himalayan vultures in Yushu City, Qinghai Province, China. Based on physical appearance and behavior based on field observation, all wild Himalayan vultures were considered healthy. Feces samples were collected immediately after defecation and transported to the laboratory in liquid nitrogen.

### 2.3. Viral particles concentration, DNA extraction, and metagenomic sequencing

The fecal samples were subjected to a process of removing potential impurities, such as host cells, bacteria, and undigested food residue, followed by the concentration of viral particles, following previously described methods (Vibin et al., 2018; Sarker, 2021), with minor modifications. The feces were aseptically resuspended and homogenized in sterile phosphate-buffered saline (PBS) at a volume ratio of 1:5. After three rounds of freeze-thawing, the samples were centrifuged at 12,000 × *g* for 5 min at 4°C to remove the precipitate. The supernatant was filtered using a 0.45 μm + 0.22 μm filter membrane and transferred to an ultracentrifugation tube containing 28% (w/w) sucrose. The mixture was then ultracentrifuged at 160,000 × *g* for 2 h at 4°C using the HIMAC CP 100wx ultracentrifuge (Hitachi, Tokyo, Japan). The supernatant was discarded, and the pellet was resuspended in 200 μL of sterile PBS. To digest unprotected nucleic acid, the resuspended solutions were nuclease-treated with a mixture of DNases (Turbo DNase, Ambion; Baseline-ZERO, Epicenter; benzonase, Novagen) and RNase (Fermentas) and incubated at 37°C for 2 h. This nuclease reaction was inactivated using 2 μL of 500 mM ethylenediaminetetraacetic acid at 75°C for 10 min. Total nucleic acid enrichment was performed using a QIAamp Viral DNA Minikit (Qiagen, Germany) according to the manufacturer’s protocol. The enriched nucleic acid samples underwent whole genome amplification with a Qiagen kit (150,054 REPLI-g Cell WGA & WTA Kit). The purified amplification products were checked for quantity and quality using a NanoDrop spectrophotometer (Thermo Fisher Scientific, Waltham, MA, United States) and 1.5% agarose electrophoresis. Six sequencing libraries (three libraries per group, with each library constructed using DNA from one sample) were generated, each containing two samples from different groups. The NEB Next® Ultra^™^ DNA Library Prep Kit for Illumina^®^ (New England Biolabs, MA, United States) was used to construct the libraries following the manufacturer’s recommendations. Index codes were added to each library. The quality and quantity of the prepared libraries were assessed using the Qubit^®^ dsDNA HS Assay Kit (Life Technologies, Grand Island, NY, United States) and Agilent 4,200 system (Agilent, Santa Clara, CA, United States). All libraries were sequenced on an Illumina NovaSeq 6,000 platform with 150 bp paired-end reads.

### 2.4. Viral metagenomic analysis

The raw paired-end reads were subjected to trimming and quality control using Trimmomatic (Bolger et al., 2014) with the following parameters: SLIDINGWINDOW:4:15 MINLEN:75 (v0.40). The trimmed reads were then aligned against the ribosome database (Silva.132) and the Himalayan vulture genome (*G. himalayensis*; accession number: GWHBAOP00000000; http://bigd.big.ac.cn/gwh) using BWA software (v0.7.17; parameter: mem –k 30) (Li and Durbin, 2009) to remove likely host DNA contamination. The resulting high-quality reads were assembled using Megahit using the parameter “--min-contig-len 200” (Li et al., 2015). Assembly sequences with a length > 2,000 bp were selected for virus identification through VirFinder (parameters: score > 0.7; *p*-value <0.05) (Ren et al., 2017), VirSorter2 (Guo et al., 2021), and IMG/VR (Integrated Microbial Genomes; https://img.jgi.doe.gov/cgi-bin/vr/main.cgi). Viral operational taxonomic units (vOTUs) were clustered using Mummer software to compare the candidate viral contigs with >95% similarity and > 85% coverage of the total sequence length. The longest representative contig within each cluster was considered a vOTU (Roux et al., 2019). CheckV was used to determine the novelty and completeness of vOTUs (Nayfach et al., 2021). To calculate the relative abundance of viral populations in each sample, clean reads were mapped to vOTUs using bowtie2 (Langmead and Salzberg, 2012) and counted using SAMtools (v1.9) (Li et al., 2009). The relative abundance of viral populations was calculated as transcripts per million reads mapped (Li et al., 2010). The taxonomic annotation (confidence score > 0.36) and host prediction (confidence score > 0.5) of VOTUs were conducted using VPF-Class tool (Pons et al., 2021). Coding DNA sequences (CDSs) were predicted from vOTUs in each sample using metaProdigal with its default parameters (Hyatt et al., 2010). To obtain the functional annotations of the CDSs, PfamScan (https://www.ebi.ac.uk/Tools/pfa/pfamscan/; e value <1e^−5^; bit score > 40) was used to detect domains and hidden Markov models, and KEGG Ghost-KOALA^1^ was used to predict KEGG orthology pathways. To annotate the glycol metabolism–related genes and antibiotic resistance genes, CDSs were searched in BLASTP against the carbohydrate active enzymes (CAZy) database^2^ and CARD database^3^ using DIAMOND (Buchfink et al., 2015).

### 2.5. Viral community analysis

The relative abundance of viral compositions was calculated as the ratio of clean read counts against all vOTUs at different viral taxonomic levels. Alpha diversity statistics, including the observed richness index and Shannon index, were calculated at the vOTU level using the *vegan* package (v2.5.7). Nonmetric multidimensional scaling (NMDS) based on the Bray-Curtis distance was performed using permutational multivariate analysis of variance through the *adonis* function of the *vegan* package. The *adonis p*-value was calculated based on 1,000 permutations. A Sankey diagram was generated using the ggalluvial package (v0.12.3) to establish connectivity between viral families and predicted hosts.

### 2.6. Phylogenetic analysis

Phylogenetic analyzes were conducted based on the genome sequences of viruses identified in this study (vOTUs with a length ≥ 6 kb and complete genomes) and reference strains belonging to four different viral families (*Siphoviridae*, *Microviridae*, *Baculoviridae*, and *Phycodnaviridae*) downloaded from the National Center for Biotechnology Information (NCBI) GenBank database. Multiple sequence alignment was performed using MAFFT (v7.475) with its default settings, and the phylogenetic trees were constructed using maximum likelihood methods in Fasttree (2.1.10) with 1,000 bootstrap replicates. The resulting phylogenetic trees were visualized using the *ggtree* package in R.

### 2.7. Statistical analysis

All statistical analyzes and data visualization were performed in R v4.2.0 integrated into RStudio v2022.02.2 + 485. Unless otherwise stated, analysis was conducted using the one-way analysis of variance (ANOVA) tests with FDR correction to compare the wild and zoo groups, with statistical significance set at ^*^p < 0.05, ^**^p < 0.01, ^***^p < 0.001, and ^****^p < 0.0001.

### 2.8. Data availability

The raw sequencing reads used in this study are available at the NCBI Sequence Read Archive database under the accession number PRJNA908771.

## 3. Results

### 3.1. Virome data overview

Six virome libraries were high-throughput sequenced using an Illumina NovaSeq PE150 platform to explore the viral communities associated with Himalayan vultures. As shown in Supplementary Table S1, 75.97 GB of raw data (506,493,246 raw pair-end reads, with an average length of 150 bp and an average GC% of 36.33%) was generated. The Q20 value for these raw reads was >96.13%. After quality filtering and trimming of raw reads, 473,057,474 clean reads were obtained (Supplementary Table S1). Given the likelihood of contamination from the genome sequences of Himalayan vultures, 183,876,320 reads were removed from the clean reads (Supplementary Table S1). The remaining 289,181,154 clean reads were assembled into longer contigs using MEGAHIT, which yielded 551,000 contigs with a length > 200 bp (Supplementary Table S2). Of these contigs, the maximum length was 133,028–276,184 bp, and the average N50 length was 1,054 bp (range: 885–1,419 bp; Supplementary Table S2). These contigs were further assigned to a series of databases using the combination of VirSorter2, VirFinder, and IMG/VR to detect viral contigs. In total, 24,043 unique viral contigs were identified as the final set of virus sequences (Supplementary Figure S1). Statistics for the viral contigs in each sample are shown in Supplementary Table S3. In total, 22,938 unique vOTUs were retrieved by filtering out the redundant contigs. These had lengths of 2,000–276,184 bp, with an N50 length of 4,987 bp (Supplementary Table S4). CheckV was used to evaluate the completeness of vOTUs, revealing that 16.28% of vOTUs were complete and high-quality (3,734 vOTUs), whereas 59.37% were low-quality (Figure 1B) and 15.11% were shared by both groups (Figure 1C).

### 3.2. Composition and comparison of viral communities between groups

**Figure 2:**
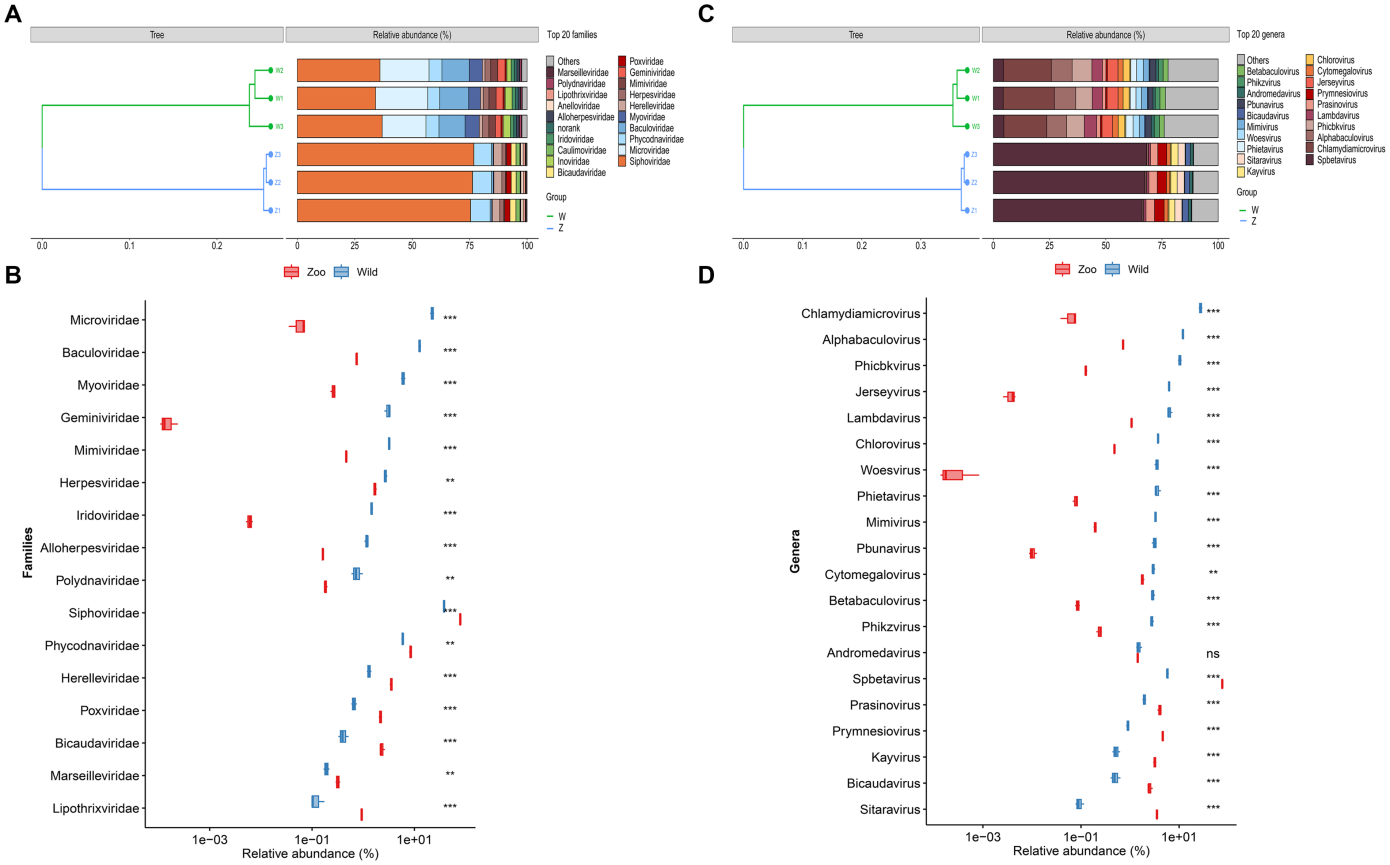
Viral community structural composition and distribution on the family and genus level. **(A)** Composition and hierarchical clustering of gut virome at the family level. **(B)** Boxplot shows the differential viral families when compared between the wild and zoo groups. **(C)** Composition and hierarchical clustering of gut virome at the genus level. **(D)** Boxplot shows the differential viral general when compared between the wild and zoo groups.

Taxonomic classification was assigned using VPF-Class, revealing 41 different viral families and 140 genera among the samples (Supplementary Table S5). To determine the relative abundance of viruses, clean reads were mapped to the viral contigs in each sample. The top ten viral families in wild Himalayan vultures were *Siphoviridae* (35.63%), *Microviridae* (21.04%), *Baculoviridae* (11.96%), *Myoviridae* (5.68%), *Phycodnaviridae* (5.51%), *Mimiviridae* (3.02%), *Geminiviridae* (2.87%), *Inoviridae* (2.59%), *Herpesviridae* (2.58%), and *Iridoviridae* (1.38%) (Figure 2A). In the zoo group, the viral communities at the family level included *Siphoviridae* (76.12%), *Phycodnaviridae* (8.13%), *Herelleviridae* (3.43%), *Bicaudaviridae* (2.27%), *Poxviridae* (2.11%), *Caulimoviridae* (1.70%), *Herpesviridae* (1.64%), *Anelloviridae* (1.03%), *Lipothrixviridae* (0.90%), and *Baculoviridae* (0.73%) (Figure 2A). Compared with the wild Himalayan vultures, the captive group showed a significant increase in *Siphoviridae*, *Phycodnaviridae*, *Herelleviridae*, *Poxviridae*, and *Bicaudaviridae*, among other viral families (Figure 2B). Conversely, the wild group had higher abundances of *Microviridae*, *Baculoviridae*, *Myoviridae*, *Herpesviridae*, *Iridoviridae*, and other families compared with the zoo group (Figure 2B).

The most abundant viral genera in the wild group were *Chlamydiamicrovirus* (21.04%), *Alphabaculovirus* (9.23%), *Phicbkvirus* (7.97%), *Lambdavirus* (4.94%), *Jerseyvirus* (4.79%), *Spbetavirus* (4.50%), *Chlorovirus* (2.85%), *Phietavirus* (2.80%), *Woesvirus* (2.72%), and *Mimivirus* (2.53%) (Figure 2C). In the zoo group, the viral genera found at relatively high abundances included *Spbetavirus* (67.23%), *Prymnesiovirus* (4.14%), *Prasinovirus* (3.56%), *Sitaravirus* (3.13%), *Kayvirus* (2.84%), *Bicaudavirus* (2.27%), *Cytomegalovirus* (1.59%), and *Andromedavirus* (1.27%) (Figure 2C). The captive Himalayan vultures harbored a higher abundance of *Spbetavirus*, *Prasinovirus*, and *Prymnesiovirus*, among other genera (Figure 2D), whereas the abundances of *Chlamydiamicrovirus*, *Alphabaculovirus*, *Phicbkvirus*, *Lambdavirus*, *Jerseyvirus*, and other genera were increased in the wild group (Figure 2D).

**Figure 3:**
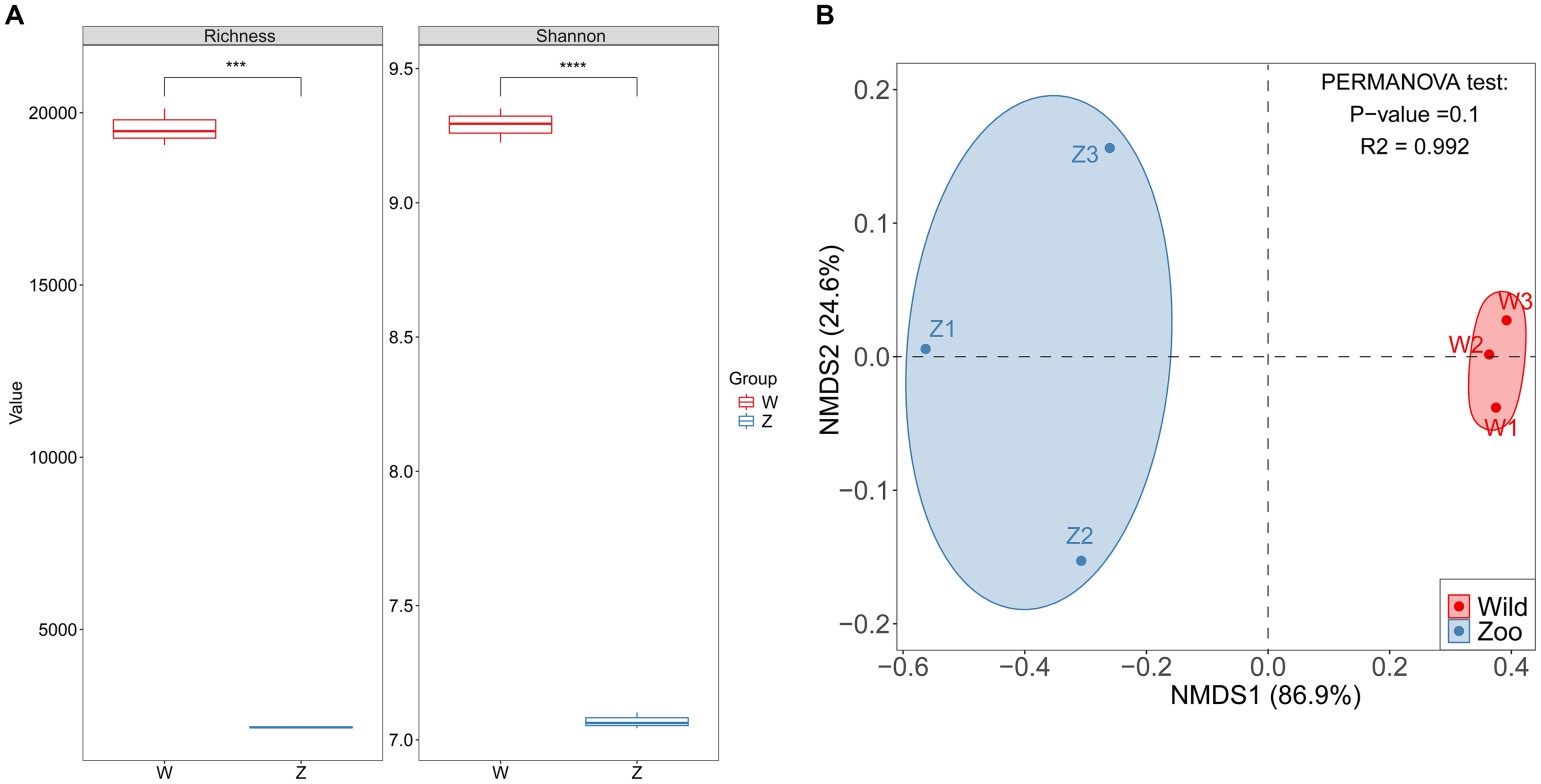
Comparison of alpha and beta diversity in gut DNA virome between the wild and zoo Himalayan vultures. **(A)** Boxplot shows the richness and Shannon index that differ between the two groups. **(B)** NMDS analysis based on Bray-Curtis distance between the two groups. The *R^2^* values and *p*-values of the *adonis* analysis are shown.

Two methods of alpha diversity, observed richness, and Shannon index, were used for sample analysis. The vOTU richness was significantly higher in the wild group compared with the zoo group (Figure 3A). Viral alpha diversity analyzes based on the Shannon index showed a significant decrease in the zoo group compared with wild group (Figure 3A). Additionally, beta diversity analyzes based on NMDS were performed to better understand the differences in viral communities between the two groups, with results indicating a clear separation between the zoo and wild groups along the first axis (Figure 3B).

### 3.3. Functional analysis of DNA virome

**Figure 4:**
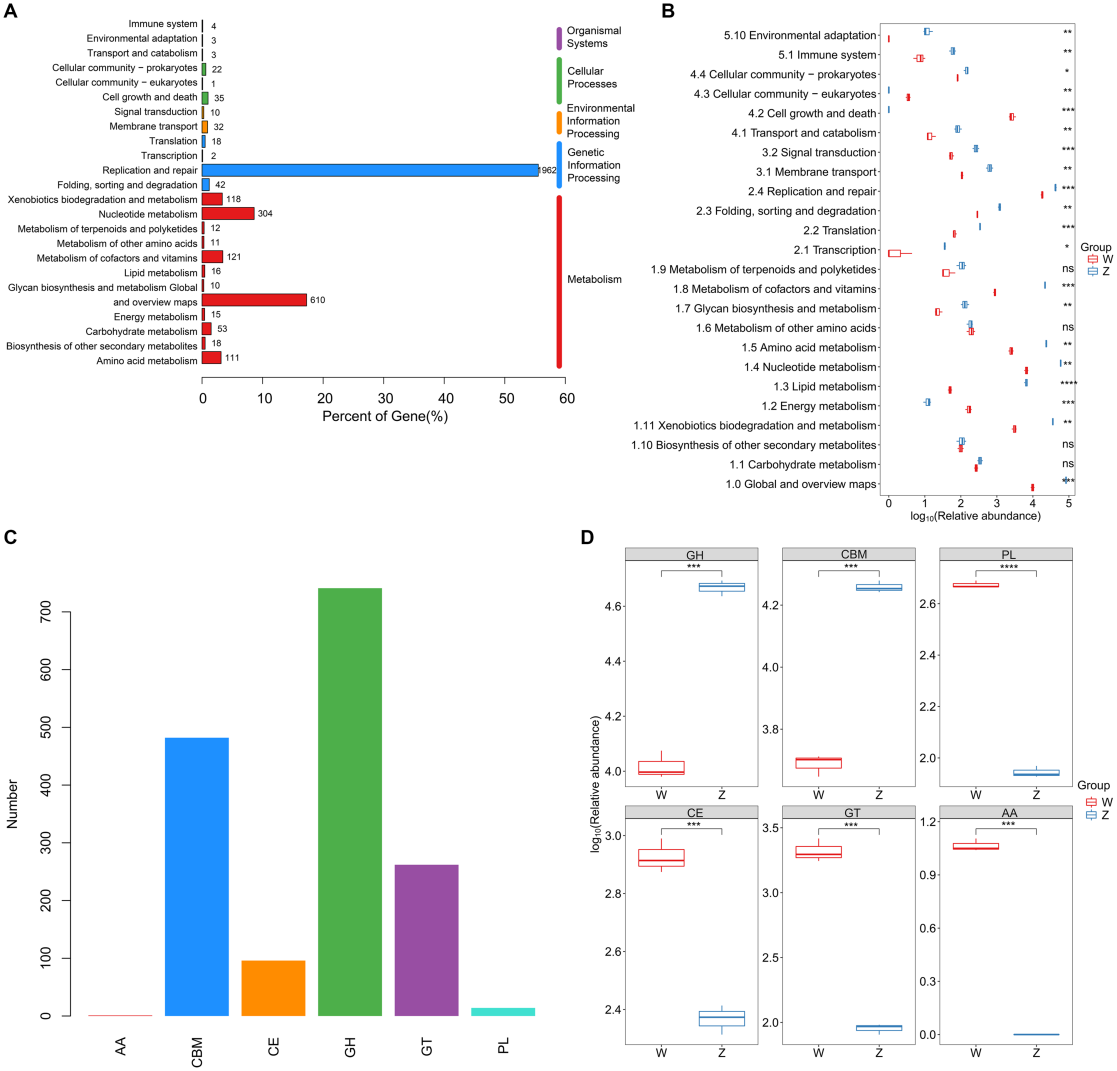
Comparison of DNA viral functions between the wild and zoo Himalayan vultures. **(A)** Composition of viral functional categories at the KEGG pathway level. **(B)** Boxplot shows the KEGG pathways that differed in abundance between the two groups. **(C)** Number of detected CAZymes. AA, auxiliary activity; CBM, carbohydrate-binding module; CE, carbohydrate esterase; GH, glycoside hydrolase; GT glycosyltransferase; PL, polysaccharide lyase. **(D)** Boxplot shows the CAZymes that differed in abundance between the two groups.

Using metaProdigal, 154,354 putative protein CDSs were identified in the six viromes of Himalayan vultures, with an average of 1.6 genes per kilobase (Supplementary Table S6). Out of these, 49,412 CDSs (32.01%) were successfully annotated against any of the five databases (NR, GO, COG, KEGG, and SWISS) (Supplementary Figure S2; Supplementary Table S7). KEGG analysis revealed the annotation of 3,533 KOs, distributed across 93 pathways (Figure 4). The functional categories associated with metabolism and genetic information processing were dominant in Himalayan vultures’ viromes (Figure 4A). The top five major categories of metabolism were nucleotide metabolism, metabolism of cofactors and vitamins, xenobiotics biodegradation and metabolism, amino acid metabolism, and carbohydrate metabolism (Figure 4A). Under genetic information processing, replication and repair was the largest category (Figure 4A). Among the 24 KEGG pathways (level 2), viral-encoded functions in the wild group were significantly enriched in cell growth and death, cellular community – eukaryotes, and energy metabolism, whereas the viral communities of the zoo group showed significant enrichment in 17 other pathways (Figure 4B).

In total, 1,596 carbohydrate-active enzymes (CAZymes) were detected in the viral genes of Himalayan vultures, including 741 glycoside hydrolases, 482 carbohydrate-binding modules, 262 glycosyltransferases, 96 carbohydrate esterases, 14 polysaccharide lyases, and 1 auxiliary activity (Figure 4C). Glycoside hydrolases and carbohydrate-binding modules were more abundant in the zoo group, whereas other CAZymes were more abundant in the wild group (Figure 4D).

Moreover, 34 unique antibiotic resistance genes (ARGs) were identified in the viral communities of Himalayan vultures. These ARGs were mainly associated with fluoroquinolone resistance (*gyrA*, *gyrB*, and *parE*), macrolide–lincosamides–streptogramin resistance (*lnuC*, *lnuD*, *lnuE*, *mphM*, *efrA*, *patB*, and *TaeA*), elfamycin resistance (*ef-Tu*), and mupirocin resistance (*mupA* and *mupB*) (Supplementary Table S8).

### 3.4. The prediction of virus-host pairs

**Figure 5:**
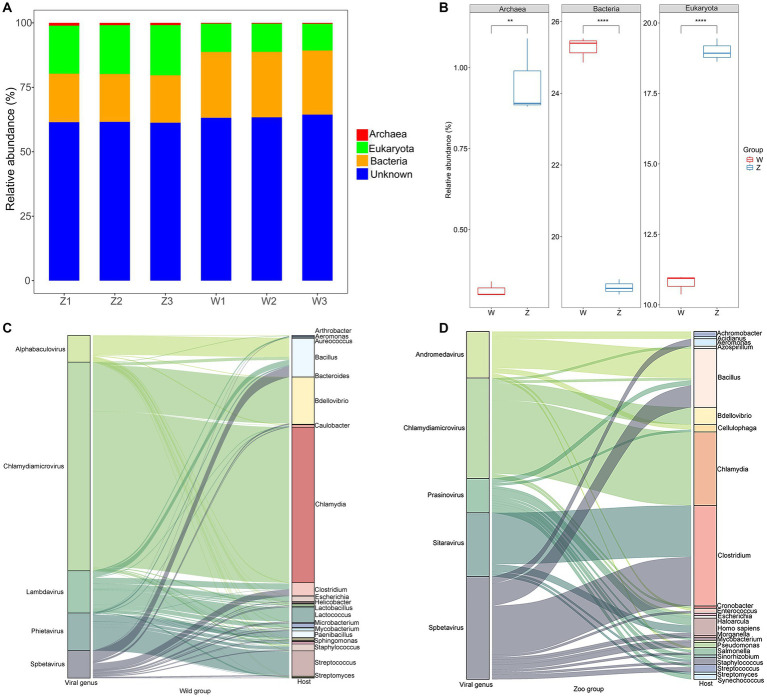
Host predictions of the viral Operational Taxonomic Units (vOTUs). **(A)** Stacked bar graph of the vOTUs classification at different taxonomic ranks by their assigned homology results of host information. **(B)** Boxplot shows the taxonomies that differed in abundance between the two groups. Sankey diagrams shows the connections between the top five viral families and their potential hosts in the wild group **(C)** and the zoo group **(D)**.

The vOTUs were assigned to putative hosts (Figure 5A). Approximately 21.93% of vOTUs were linked to bacterial hosts, whereas 14.88% of vOTUs were predicted to have eukaryotic hosts, suggesting potential viral infection transmission across domains (Figure 5A). In the zoo group, more vOTUs were linked to eukaryotic and archaeal hosts, whereas a larger number of abundant vOTUs were assigned to bacterial hosts in the wild group (Figure 5B). Sankey diagrams revealed the connections between the top five viral families with the highest number of infection hosts and their potential hosts (Figures 5C,D). In the wild group, the most common hosts for the viromes of Himalayan vultures were *Chlamydia*, *Bacillus*, *Bdellovibrio*, *Streptococcus*, *Lactococcus*, and *Clostridium* (Figure 5C). In the zoo group, the genera *Bacillus*, *Clostridium*, *Chlamydia*, *Homo*, and *Bdellovibrio* were found to be the most common hosts for the Himalayan vultures (Figure 5D).

### 3.5. Phylogenetic analyzes of viral sequences in wild Himalayan vultures

**Figure 6:**
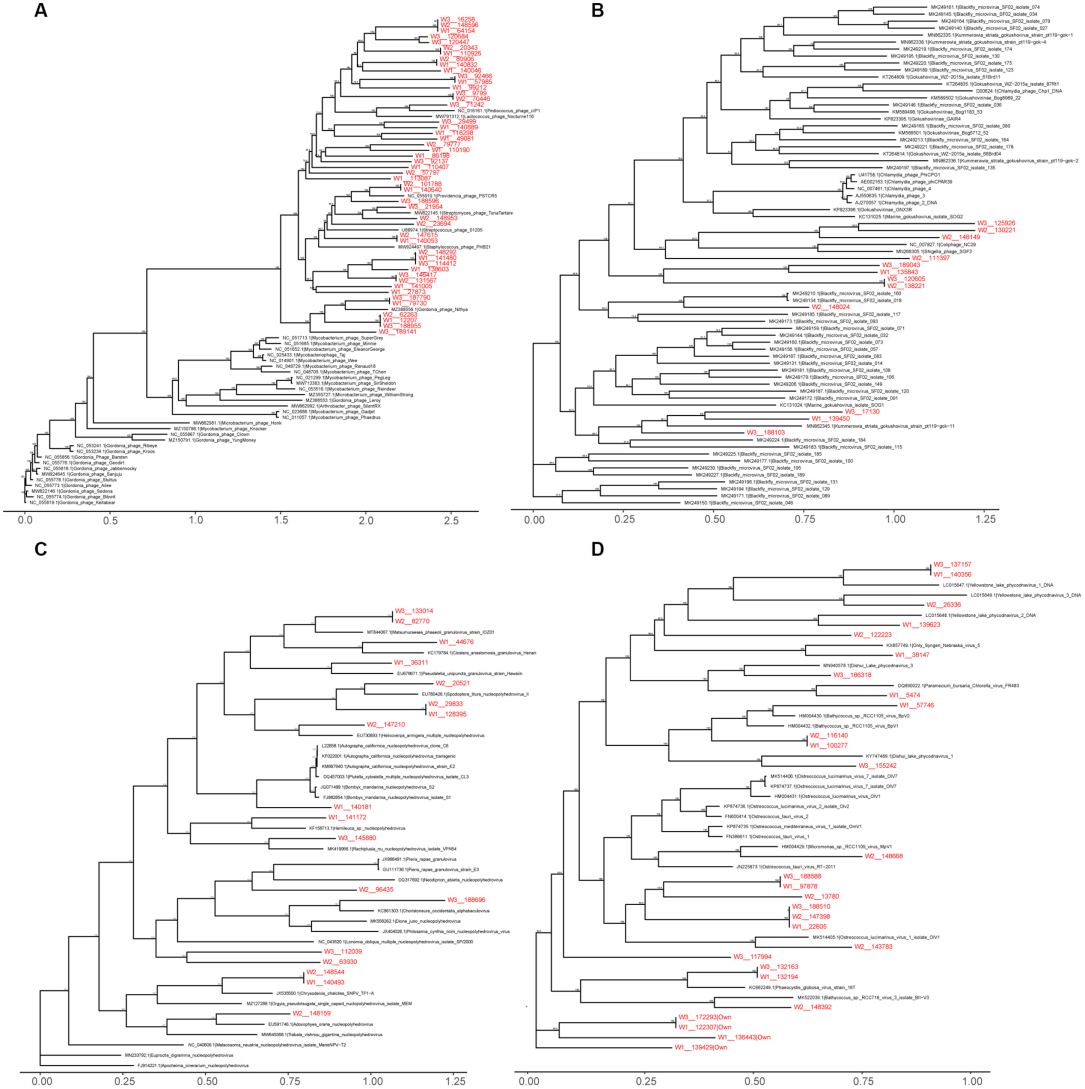
Maximum likelihood phylogenetic tree of *Siphoviridae* **(A)**, *Microviridae* **(B)**, *Baculoviridae* **(C)**, *Phycodnaviridae* **(D)**. Virus names are followed by the GenBank accession numbers. The viral Operational Taxonomic Units (vOTUs) identified in the present study are marked with red color. The numbers beside the branches represent statistical confidence in clades based on 1,000 bootstrap replicates, only bootstrap values ≥90% are shown.

Within the viral family *Siphoviridae*, which includes a total of 47 genera with double-stranded DNA (dsDNA) genomes of approximately 50 kb encoding around 70 genes, we identified 2,475 vOTUs belonging to 38 genera in the wild Himalayan vultures. Among them, seven genera (*Lambdavirus*, *Spbetavirus*, *Phietavirus*, *Andromedavirus*, *Likavirus*, *Phicbkvirus*, and *Biseptimavirus*) contained more than 100 vOTUs. Phylogenetic analysis (Figure 6A) revealed that these unique vOTUs were closely related and showed some minor genetic overlap with the current viral database. The viral family *Microviridae*, which encompasses six genera with single-stranded DNA genomes of approximately 4.4–6.1 kb, comprised 1,429 vOTUs in the wild Himalayan vultures. Phylogenetic analysis indicated that these vOTUs shared some similarities with *blackfly_microvirus* (Figure 6B). The viral family *Baculoviridae*, consisting of four genera with dsDNA genomes of approximately 80–180 kb encoding 100–180 genes, contained 374 vOTUs in the wild Himalayan vultures. These vOTUs exhibited similarities with *nucleopolyhedrovirus* and *granulovirus* (Figure 6C). Lastly, the viral family *Phycodnaviridae*, which includes six genera with dsDNA genomes of approximately 100–560 kb encoding around 700 genes, contained 489 vOTUs belonging to five genera in the wild Himalayan vultures. Phylogenetic analysis revealed similarities with *phycodnavirus* (Figure 6D).

## 4. Discussion

In the past decade, advancements in next-generation sequencing technologies and bioinformatic analysis of viral metagenomes have greatly facilitated the study of viromes in diverse wildlife species (Mokili et al., 2012; Li et al., 2021). These methods have allowed for the characterization of both known and unknown viruses in various wildlife species, including mosquitoes, ticks, bats, and rodents (Zhou et al., 2022). Among these species, wild birds exhibit high biodiversity and serve as significant reservoirs of different viruses, such as avian influenza virus, Newcastle disease virus, and West Nile virus which have the potential for cross-species transmission, posing a serious threat to human health (Barroso et al., 2021) especially considering their migration, and flocking behavior. Despite the growing body of research on the bacteriome of birds, our understanding of bird viromes remains limited (François and Pybus, 2020; Tyson-Pello and Olsen, 2020). Only a few previous studies have explored bird viromes, providing valuable insights into the composition, function, and evolution of avian viruses. For instance, Shan et al. (2022) conducted an extensive investigation of viral communities in cloacal swabs collected from 3,182 wild and breeding birds belonging to 10 different orders within the Aves class across eight provinces in China, resulting in the identification of 707 viral genomes. This large-scale study significantly expanded our knowledge of avian viral diversity. Although most avian virus studies have focused on birds from the orders *Anseriformes* and *Galliformes*, which are closely related to common domestic poultry (Ramírez-Martínez et al., 2018; Vibin et al., 2020), as well as resident birds in the order *Passeriformes* (Truchado et al., 2020), there is a lack of information regarding viral diversity, evolution, and ecology in scavenging birds from the Qinghai-Tibetan Plateau, particularly vulture species (Zhu et al., 2022). Therefore, investigating the viromes of understudied Himalayan vulture populations can contribute to the prevention of potential viral outbreaks in the future, given their feeding habits that involve consuming the carcasses of various wild and domestic animals on the plateau. In this study, we conducted the first comprehensive analysis of the gut DNA viromes in Himalayan vultures using viral metagenomics, aiming to explore their composition, function, and the differences between wild and captive individuals.

Keeping birds in captivity in zoos represents a drastic departure from their natural living environment, which can have profound effects on their gut bacterial diversity. Previous studies have demonstrated a significant reduction in gut bacterial diversity in captive birds compared with their wild counterparts (Oliveira et al., 2020; Wu et al., 2021). Consistent with these findings, our study revealed a noteworthy decrease in the richness and diversity of viruses in the captive groups, consistent with the previous investigations of the gut bacteriome. Captive birds undergo various changes that likely impact their gut microbiome, including alterations or limitations in diet, antibiotic treatments, reduced exposure to diverse microbial communities from different habitats, and heightened contact with human-associated microbes (McKenzie et al., 2017). The diet provided to the Himalayan vultures in the zoo was consistent and hygienic, and the relatively confined living area restricted their exposure to microbes, potentially explaining the lower diversity observed in their gut viromes. Due to small sample sizes, the PERMANOVA *p*-value for beta-diversity was not significant. The resulting NMDS plot appeared to show that the high degree of separation between the two groups across the first axis. Changes in beta-diversity further supported the notion of stable gut viral communities in captive individuals, distinct from those observed in the wild group. Unsurprisingly, the shared diet, and living environment contribute to the higher similarity in gut viromes within the zoo group. Conversely, free-living individuals from the wild group, with their expansive territories, and diverse diets, exhibited a certain degree of variation in their gut viromes.

In line with the prevalence of bacteria in the gut microbiome, gut viral communities appear to be primarily composed of prokaryotic viruses (Dion et al., 2020). In this study, 41 viral families and 140 viral genera were identified, primarily consisting of bacteriophages. Within the wild group, 64.94% of gut DNA virome sequences exhibited homology to phages from the *Siphoviridae*, *Microviridae*, *Myoviridae*, and *Inoviridae* families. In the zoo group, 79.55% of gut DNA virome sequences showed homology to phages from the *Siphoviridae* and *Herelleviridae* families. Bacteriophages, the apex predators of the bacterial realm, were found to be the most abundant group of viruses in the gut virome of humans and other animals (Liang and Bushman, 2021; Harvey and Holmes, 2022). Bacteriophages can influence the structure of the gut bacteriome and maintain high levels of microbial community diversity through kill-the-winner dynamics (Maslov and Sneppen, 2017). We hypothesized that bacteriophages likely play a vital role in modulating the bacterial community in the gut of Himalayan vultures, particularly the dominant microbial members of the gut microbiota. However, in our study, we solely predicted the putative hosts of the viral contigs and did not sequence the bacteriome. The interactions between bacteriophages and bacteria are intricate and challenging to predict owing to the considerable genetic diversity present in bacterial and bacteriophage populations. Therefore, future investigations should incorporate experimental evidence to elucidate these interactions and enhance our understanding of the impact of phages within the gastrointestinal environment.

In the wild group, a certain proportion of viral sequences exhibited homology with animal viruses from the *Mimiviridae* and *Herpesviridae* families, plant viruses from the *Phycodnaviridae* and *Geminiviridae* families, and insect viruses from the *Baculoviridae* and *Iridoviridae* families. In the zoo group, a certain proportion of viral sequences displayed homology with animal viruses from the *Poxviridae*, *Herpesviridae*, and *Anelloviridae* families, plant viruses from the *Phycodnaviridae* and *Caulimoviridae* families, insect viruses from the *Baculoviridae* family, and archaea viruses from the *Bicaudaviridae* and *Lipothrixviridae* families. These findings highlight the high diversity of viral communities in Himalayan vultures, and the presence of animal, plant, and insect viruses often reflects their acquisition from the host’s diet and living environment. As scavenger birds, wild Himalayan vultures consume numerous bacteria and viruses present in decaying food. Although this could explain the origin of these gut viruses, it remains unclear whether the viruses selected and retained by the host during the long evolutionary process can fulfill beneficial physiological functions or protective roles similar to those played by gut commensal bacteria, and this matter requires further research for confirmation. In captive Himalayan vultures, the diversity and abundance of the gut microbiota undergo changes due to dietary restrictions and the captive living environment. Thus, changes in the composition and abundance of the gut virome may result from direct or indirect effects associated with changes in the gut microbiota between wild and captive states.

The functional capacity of the gut virome was characterized by the identification of 93 KEGG pathways, which exhibited a high enrichment in metabolism and genetic information processing. This finding aligns with the predicted function of the gut bacteriome in Himalayan vultures, as previously reported by our research group (Wang et al., 2021), in which metabolism (47.30%) and genetic information processing (18.93%) ranked in the first two categories. Metabolism, as the most prevalent functional category, likely signifies a high metabolic activity within the viral communities. This could be attributed to the collection of fecal samples after the Himalayan vultures had completed their feeding. Another notable functional category is genetic information processing, particularly the replication and repair pathway, which may be induced by increased exposure to harmful substances from carrion-borne pathogens. Furthermore, a diverse array of AMGs, including CAZymes and ARGs, were identified in the gut virome of Himalayan vultures. The presence of these AMGs in viruses is presumed to enhance host metabolism and facilitate the generation of new viruses. In our study, we observed a significant presence of viral CAZymes, including core hydrolysis enzymes essential for the degradation of complex polysaccharides. These results were in line with our previously published prediction of gut microbiota function in Himalayan vultures, where carbohydrate metabolism (22.97%) was the highest subtype under the metabolic category (Wang et al., 2021). We speculate that these viral CAZymes might participate in the decomposition and utilization of complex carbohydrates, thereby promoting viral replication within the gut ecosystem of Himalayan vultures. Notably, both the bacteriome and virome in bird guts are considered reservoirs of ARGs. The presence of ARGs encoded by gut viruses in Himalayan vultures suggests their potential involvement in the carriage and transmission of functional ARGs, although further investigation is required to confirm this hypothesis.

This study includes several limitations that provide a direction for future research. First, the small sample size is an obvious limitation, primarily because collecting Himalayan vultures feces in both wild and captive settings is challenging. Fresh feces are difficult to collect given the irregularity of excretion from the birds. Virome sequencing is also expensive, which is another objective reason for the low sample size of this study. In future works, a larger sample size of Himalayan vultures and broader geographic area coverage, including more wild environments, are needed. Second, although many software and approaches have been developed for viromes analyzes, the process from sample collection, sequencing preparation to bioinformatics analysis is challenging, and can lead to biases. For example, many viruses occur as integrated prophages in the genomes of bacteria. In our study, the enrichment of virus-like particles before high-throughput sequencing can reduce the background noise of cellular organisms and enrich extracellular viruses to a certain extent, but unfortunately cannot capture intracellular viruses such as phages. Identifying the host range of viral sequences is also essential for understanding gut viral communities as a whole. Computational methods used to predict bacteriophage hosts fall into two categories: sequence alignment-dependent or-independent. Alignment-dependent methods are usually performed by screening for CRISPR spacers matching to phages. However, only about 40% of bacteria carry a CRISPR-Cas system (Horvath and Barrangou, 2010). Alignment-independent tools tend to exhibit relatively low accuracy (Ahlgren et al., 2017). In this study, VPF-Class, as the alignment-dependent method, was used to predict the hosts of viral contigs based on the assignment of their proteins to a set of classified viral protein families. In the future, we anticipate that alignment-independent and alignment-based approaches will be integrated to improve the overall sensitivity of host assignments without compromising accuracy. Lastly, the lack of sufficient virome databases limits our ability to identify additional viruses that may be present in our viral sequencing data, which can only be addressed by significantly expanding the virome sequences and annotations in existing databases. The limitations above do not affect the robustness of the current results, but follow-up studies in a larger sample sizes will still complement some of the shortcomings of the current study and provide additional new findings.

## 5. Conclusion

In conclusion, our study provides initial insights into the DNA viral composition of the gut virome in Himalayan vultures. The comparative analysis of viromes enhances our understanding of viral communities in wild and captive Himalayan vultures. However, future studies should validate these findings using cultivation-based whole-genome sequencing. Additionally, broader sampling from different locations is necessary to enhance our understanding of the viral diversity present in the gastrointestinal tracts of this scavenger bird species.

## Data availability statement

The datasets presented in this study can be found in online repositories. The names of the repository/repositories and accession number(s) can be found in the article/Supplementary material.

## Ethics statement

The animal study was reviewed and approved by this animal study was reviewed and approved by the Ethical Committee of Qinghai University.

## Author contributions

CL and WW designed the experiments. YW, BT, SZ, SH, and WZ completed the field sampling. JZ, HC, JL, FL, and YB performed the data analysis and prepared the figures. JZ and WW wrote the manuscript. ZL, KS, CL, and WW contributed to the revision of manuscript. All authors contributed to the article and approved the submitted version.

## Funding

This research was funded by the National Natural Science Foundation of China (grant No. 31960277); the National Natural Science Foundation of China and Russian Foundation for Basic Research Cooperative Exchange Project (grant No. 32111530018, 21-54-53031); the Program of Science and Technology International Cooperation Project of Qinghai Province (grant No. 2022-HZ-812).

## Conflict of interest

The authors declare that the research was conducted in the absence of any commercial or financial relationships that could be construed as a potential conflict of interest.

## Publisher’s note

All claims expressed in this article are solely those of the authors and do not necessarily represent those of their affiliated organizations, or those of the publisher, the editors and the reviewers. Any product that may be evaluated in this article, or claim that may be made by its manufacturer, is not guaranteed or endorsed by the publisher.

## Footnotes

^1^ https://www.kegg.jp/ghostkoala/

^2^ http://www.cazy.org/

^3^ https://card.mcmaster.ca/
