# Supplementary material for: Comparative analysis of gut DNA viromes in wild and captive Himalayan vultures
Source: Front Microbiol. 2023 Aug 2;14:1120838. doi: 10.3389/fmicb.2023.1120838 (PMC10433386; doi:10.3389/fmicb.2023.1120838)
Supplement: Supplementary file 1 [file Data_Sheet_1.docx]

Supplementary Material

# Supplementary Figures and Tables

## Supplementary Figures


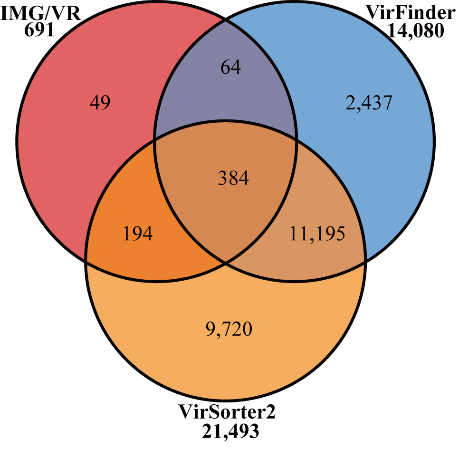


**Supplementary Figure 1.** Venn diagram of shared and unique viral contigs identified with VirSorter2, VirFinder, and IMG/VR databases.


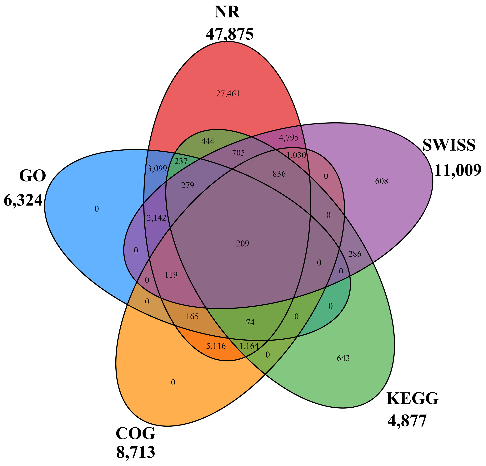


**Supplementary Figure 2.** Venn diagram of putative protein coding sequence annotated against the five databases (NR, GO, COG, KEGG and SWISS).

## Supplementary Tables

**Supplementary Table 1. Overview of the raw reads and clean reads of each sample.**

| Samples | Raw_base (G) | Raw_reads | GC Content (%) | Q20 (%) | Clean_base (G) | Clean_reads | Clean reads after host subtraction |
| --- | --- | --- | --- | --- | --- | --- | --- |
| Z1 | 12.40 | 82,656,066 | 31.10 | 96.13 | 11.54 | 77,441,758 | 39,725,722 |
| Z2 | 13.75 | 91,693,660 | 31.03 | 96.20 | 12.84 | 86,160,320 | 44,485,394 |
| Z3 | 13.68 | 91,189,870 | 31.07 | 96.24 | 12.73 | 85,434,230 | 38,132,490 |
| W1 | 12.48 | 83,211,790 | 41.09 | 96.31 | 11.57 | 77,645,296 | 55,472,226 |
| W2 | 12.29 | 81,961,810 | 41.85 | 96.13 | 11.32 | 75,997,238 | 50,299,776 |
| W3 | 11.37 | 75,780,050 | 41.83 | 96.14 | 10.48 | 70,378,632 | 61,065,546 |
| Total | 75.97 | 506,493,246 | - | - | 70.48 | 473,057,474 | 289,181,154 |

**Supplementary Table 2. Overview of the assembly data of each sample.**

| Samples | Total_base (Mb) | Total number | Maximum length (bp) | Minimum length (bp) | N50 (bp) |
| --- | --- | --- | --- | --- | --- |
| Z1 | 24.41 | 29,269 | 143,932 | 200 | 970 |
| Z2 | 19.97 | 20,262 | 137,453 | 200 | 1,419 |
| Z3 | 20.11 | 21,680 | 133,028 | 200 | 1,219 |
| W1 | 114.91 | 141,502 | 140,097 | 200 | 942 |
| W2 | 116.60 | 149,105 | 276,184 | 200 | 889 |
| W3 | 147.33 | 189,182 | 135,385 | 200 | 885 |
| Total | 443.33 | 551,000 | - | - | - |

**Supplementary Table 3.** The statistics of viral contigs detected using the combination of VirSorter2, VirFinder, and IMG/VR.

| Methods | Z1 | Z2 | Z3 | W1 | W2 | W3 |
| --- | --- | --- | --- | --- | --- | --- |
| IMG/VR | 52 | 47 | 54 | 173 | 153 | 212 |
| VirFinder | 428 | 434 | 421 | 4,090 | 3,769 | 4,938 |
| VirSorter2 | 674 | 658 | 621 | 6,280 | 5,817 | 7,443 |

**Supplementary Table 4.** Basic information about viral Operational Taxonomic Units (vOTUs) in each sample.

| Samples | Number of vOTUs | Total length (bp) | Average length (bp) | Maximum length (bp) | Minimum length (bp) | GC Content (%) |
| --- | --- | --- | --- | --- | --- | --- |
| Z1 | 684 | 4,262,397 | 6,232 | 143,932 | 2,006 | 36.09 |
| Z2 | 672 | 3,927,026 | 5,844 | 137,453 | 2,000 | 35.55 |
| Z3 | 629 | 4,036,674 | 6,418 | 133,028 | 2,001 | 35.86 |
| W1 | 6,732 | 28,061,612 | 4,168 | 140,097 | 2,000 | 43.12 |
| W2 | 6,165 | 25,663,132 | 4,163 | 276,184 | 2,000 | 43.71 |
| W3 | 8,056 | 33,191,605 | 4,120 | 135,385 | 2,000 | 43.73 |
| Total | 22,938 | 99,142,446 | - | - | - | - |

**Supplementary Table 5.** Numbers of different viral taxonomy (from family to genus) in each sample.

| Samples | Family | Genus |
| --- | --- | --- |
| Z1 | 33 | 87 |
| Z2 | 33 | 92 |
| Z3 | 36 | 98 |
| W1 | 41 | 137 |
| W2 | 41 | 137 |
| W3 | 41 | 138 |
| Total | 41 | 140 |

**Supplementary Table 6.** The statistics of coding sequence (CDS) predicted by metaProdigal.

| Type | Statistics data |
| --- | --- |
| CDS num | 154,354 |
| CDS total length | 89,567,403 bp |
| CDS density | 1.556 genes per kb |
| CDS average length | 580.27 bp |
| Intergenetic region length | 9,575,043 bp |
| CDS/Genome(coding percentage) | 90.30% |
| Intergenetic length/Genome | 9.75% |
| GC content in gene region | 41.70% |
| GC content in intergenetic region | 39.00% |

**Supplementary Table 7.** Summary of protein coding sequence (CDS) annotated by five databases.

| Database | Total_unigenes | Annoted_unigenes | Percent |
| --- | --- | --- | --- |
| NR | 154,354 | 47,875 | 31.02% |
| GO | 154,354 | 6,324 | 4.10% |
| COG | 154,354 | 8,713 | 5.64% |
| KEGG | 154,354 | 4,877 | 3.16% |
| SWISS | 154,354 | 11,009 | 7.13% |
| In all database | 154,354 | 209 | 0.14% |
| At least one database | 154,354 | 49,412 | 32.01% |

**Supplementary Table 8.** Detailed information of the detected antibiotic resistance genes.

| Viral geneID | Similarity (%) | GeneID | Species | Drug class | Resistance mechanism |
| --- | --- | --- | --- | --- | --- |
| Z1__11207_1 | 90.35 | efrA | Enterococcus faecium | macrolide antibiotic;fluoroquinolone antibiotic;cephalosporin;penam;tetracycline antibiotic;peptide antibiotic;acridine dye;rifamycin antibiotic;pleuromutilin antibiotic;nitroimidazole antibiotic | antibiotic efflux |
| Z1__11207_2 | 97.63 | patB | Streptococcus pneumoniae TIGR4 | macrolide antibiotic;fluoroquinolone antibiotic;cephalosporin;penam;tetracycline antibiotic;peptide antibiotic;acridine dye;rifamycin antibiotic;pleuromutilin antibiotic;nitroimidazole antibiotic | antibiotic efflux |
| Z1__11829_112 | 97.33 | Mycobacterium tuberculosis thyA with mutation conferring resistance to para-aminosalicylic acid | Mycobacterium tuberculosis H37Rv | para-aminosalicylic acid | antibiotic target alteration |
| Z1__14087_1 | 99.39 | lnuE | synthetic construct | lincosamide antibiotic | antibiotic inactivation |
| Z1__19760_4 | 100 | Ureaplasma urealyticum gyrB conferring resistance to fluoroquinolone | Ureaplasma urealyticum serovar 10 str. ATCC 33699 | fluoroquinolone antibiotic;aminocoumarin antibiotic | antibiotic target alteration |
| Z1__19760_5 | 100 | Staphylococcus aureus gyrA conferring resistance to fluoroquinolones | Staphylococcus aureus subsp. aureus MRSA252 | fluoroquinolone antibiotic;nybomycin | antibiotic target alteration |
| Z1__20059_1 | 63.5 | Ureaplasma urealyticum gyrB conferring resistance to fluoroquinolone | Ureaplasma urealyticum serovar 10 str. ATCC 33699 | fluoroquinolone antibiotic;aminocoumarin antibiotic | antibiotic target alteration |
| Z1__20530_1 | 94.6 | optrA | Enterococcus faecalis | macrolide antibiotic;lincosamide antibiotic;streptogramin antibiotic;tetracycline antibiotic;oxazolidinone antibiotic;phenicol antibiotic;pleuromutilin antibiotic | antibiotic target protection |
| Z1__21277_2 | 100 | Clostridioides difficile EF-Tu mutants conferring resistance to elfamycin | Clostridioides difficile | elfamycin antibiotic | antibiotic target alteration |
| Z1__21365_11 | 99.44 | Bartonella bacilliformis gyrB conferring resistance to aminocoumarin | Bartonella bacilliformis KC583 | aminocoumarin antibiotic | antibiotic target alteration |
| Z1__2391_3 | 99.39 | lnuC | Streptococcus agalactiae | lincosamide antibiotic | antibiotic inactivation |
| Z1__24543_1 | 100 | RlmA(II) | Streptococcus pneumoniae | macrolide antibiotic;lincosamide antibiotic | antibiotic target alteration |
| Z1__26683_1 | 100 | tetM | Staphylococcus aureus subsp. aureus ST398 | tetracycline antibiotic | antibiotic target protection |
| Z1__28431_21 | 99.39 | lnuD | Streptococcus uberis | lincosamide antibiotic | antibiotic inactivation |
| Z1__28431_22 | 95.7 | mphM | Bacillus thuringiensis | macrolide antibiotic | antibiotic inactivation |
| Z2__12316_11 | 87.05 | bcrA | Bacillus licheniformis | macrolide antibiotic;fluoroquinolone antibiotic;cephalosporin;penam;tetracycline antibiotic;peptide antibiotic;acridine dye;rifamycin antibiotic;pleuromutilin antibiotic;nitroimidazole antibiotic | antibiotic efflux |
| Z2__16989_2 | 99.65 | ANT(6)-Ia | Exiguobacterium sp. S3-2 | aminoglycoside antibiotic | antibiotic inactivation |
| Z2__18748_4 | 83.13 | Streptococcus pneumoniae PBP1a conferring resistance to amoxicillin | Streptococcus pneumoniae | monobactam;carbapenem;cephalosporin;cephamycin;penam | antibiotic target alteration |
| Z2__18969_2 | 100 | Clostridioides difficile EF-Tu mutants conferring resistance to elfamycin | Clostridioides difficile | elfamycin antibiotic | antibiotic target alteration |
| Z2__2551_21 | 97.33 | Mycobacterium tuberculosis thyA with mutation conferring resistance to para-aminosalicylic acid | Mycobacterium tuberculosis H37Rv | para-aminosalicylic acid | antibiotic target alteration |
| Z2__2928_1 | 88.72 | efrA | Enterococcus faecium | macrolide antibiotic;fluoroquinolone antibiotic;cephalosporin;penam;tetracycline antibiotic;peptide antibiotic;acridine dye;rifamycin antibiotic;pleuromutilin antibiotic;nitroimidazole antibiotic | antibiotic efflux |
| Z2__2928_2 | 84.46 | patB | Streptococcus pneumoniae TIGR4 | macrolide antibiotic;fluoroquinolone antibiotic;cephalosporin;penam;tetracycline antibiotic;peptide antibiotic;acridine dye;rifamycin antibiotic;pleuromutilin antibiotic;nitroimidazole antibiotic | antibiotic efflux |
| Z2__4194_4 | 99.39 | lnuE | synthetic construct | lincosamide antibiotic | antibiotic inactivation |
| Z2__4650_1 | 64.49 | efrA | Enterococcus faecium | macrolide antibiotic;fluoroquinolone antibiotic;cephalosporin;penam;tetracycline antibiotic;peptide antibiotic;acridine dye;rifamycin antibiotic;pleuromutilin antibiotic;nitroimidazole antibiotic | antibiotic efflux |
| Z2__4650_2 | 94.82 | patB | Streptococcus pneumoniae TIGR4 | macrolide antibiotic;fluoroquinolone antibiotic;cephalosporin;penam;tetracycline antibiotic;peptide antibiotic;acridine dye;rifamycin antibiotic;pleuromutilin antibiotic;nitroimidazole antibiotic | antibiotic efflux |
| Z2__6494_1 | 100 | Clostridioides difficile gyrB conferring resistance to fluoroquinolone | Clostridium ljungdahlii DSM 13528 | fluoroquinolone antibiotic;aminocoumarin antibiotic | antibiotic target alteration |
| Z2__6494_2 | 97.55 | Clostridioides difficile gyrA conferring resistance to fluoroquinolones | Clostridioides difficile 630 | fluoroquinolone antibiotic;nybomycin | antibiotic target alteration |
| Z2__8354_2 | 99.68 | Ureaplasma urealyticum gyrB conferring resistance to fluoroquinolone | Ureaplasma urealyticum serovar 10 str. ATCC 33699 | fluoroquinolone antibiotic;aminocoumarin antibiotic | antibiotic target alteration |
| Z2__8530_1 | 98.06 | Clostridioides difficile rpoB with mutation conferring resistance to rifampicin | Clostridium ljungdahlii DSM 13528 | peptide antibiotic;rifamycin antibiotic | antibiotic target alteration;antibiotic target replacement |
| Z2__9147_1 | 100 | Cutibacterium acnes gyrA conferring resistance to fluoroquinolones | Cutibacterium acnes subsp. defendens ATCC 11828 | fluoroquinolone antibiotic;nybomycin | antibiotic target alteration |
| Z2__9838_1 | 99.39 | lnuC | Streptococcus agalactiae | lincosamide antibiotic | antibiotic inactivation |
| Z3__11642_2 | 100 | Clostridioides difficile EF-Tu mutants conferring resistance to elfamycin | Clostridioides difficile | elfamycin antibiotic | antibiotic target alteration |
| Z3__13097_4 | 100 | Clostridioides difficile gyrB conferring resistance to fluoroquinolone | Clostridium ljungdahlii DSM 13528 | fluoroquinolone antibiotic;aminocoumarin antibiotic | antibiotic target alteration |
| Z3__13097_5 | 90.93 | Clostridioides difficile gyrA conferring resistance to fluoroquinolones | Clostridioides difficile 630 | fluoroquinolone antibiotic;nybomycin | antibiotic target alteration |
| Z3__15302_6 | 96.93 | lmrD | Lactococcus lactis | macrolide antibiotic;fluoroquinolone antibiotic;lincosamide antibiotic;cephalosporin;penam;tetracycline antibiotic;peptide antibiotic;acridine dye;rifamycin antibiotic;pleuromutilin antibiotic;nitroimidazole antibiotic | antibiotic efflux |
| Z3__17123_25 | 95.7 | mphM | Bacillus thuringiensis | macrolide antibiotic | antibiotic inactivation |
| Z3__17123_26 | 99.39 | lnuD | Streptococcus uberis | lincosamide antibiotic | antibiotic inactivation |
| Z3__19485_2 | 99.68 | Ureaplasma urealyticum gyrB conferring resistance to fluoroquinolone | Ureaplasma urealyticum serovar 10 str. ATCC 33699 | fluoroquinolone antibiotic;aminocoumarin antibiotic | antibiotic target alteration |
| Z3__20341_8 | 100 | Staphylococcus aureus gyrA conferring resistance to fluoroquinolones | Staphylococcus aureus subsp. aureus MRSA252 | fluoroquinolone antibiotic;nybomycin | antibiotic target alteration |
| Z3__20341_9 | 100 | Ureaplasma urealyticum gyrB conferring resistance to fluoroquinolone | Ureaplasma urealyticum serovar 10 str. ATCC 33699 | fluoroquinolone antibiotic;aminocoumarin antibiotic | antibiotic target alteration |
| Z3__20579_1 | 62.58 | efrA | Enterococcus faecium | macrolide antibiotic;fluoroquinolone antibiotic;cephalosporin;penam;tetracycline antibiotic;peptide antibiotic;acridine dye;rifamycin antibiotic;pleuromutilin antibiotic;nitroimidazole antibiotic | antibiotic efflux |
| Z3__20579_2 | 94.82 | patB | Streptococcus pneumoniae TIGR4 | macrolide antibiotic;fluoroquinolone antibiotic;cephalosporin;penam;tetracycline antibiotic;peptide antibiotic;acridine dye;rifamycin antibiotic;pleuromutilin antibiotic;nitroimidazole antibiotic | antibiotic efflux |
| Z3__5336_43 | 97.33 | Mycobacterium tuberculosis thyA with mutation conferring resistance to para-aminosalicylic acid | Mycobacterium tuberculosis H37Rv | para-aminosalicylic acid | antibiotic target alteration |
| Z3__6168_16 | 53.63 | Staphylococcus aureus gyrA conferring resistance to fluoroquinolones | Staphylococcus aureus subsp. aureus MRSA252 | fluoroquinolone antibiotic;nybomycin | antibiotic target alteration |
| Z3__8698_2 | 99.63 | ANT(6)-Ib | Campylobacter fetus subsp. fetus | aminoglycoside antibiotic | antibiotic inactivation |
| W1__106382_1 | 99.07 | Pseudomonas mutant PhoP conferring resistance to colistin | Pseudomonas aeruginosa PAO1 | macrolide antibiotic;fluoroquinolone antibiotic;cephalosporin;penam;tetracycline antibiotic;peptide antibiotic;acridine dye;rifamycin antibiotic;pleuromutilin antibiotic;nitroimidazole antibiotic | antibiotic target alteration;antibiotic efflux |
| W1__126639_1 | 99.61 | mupA | Staphylococcus aureus | mupirocin | antibiotic target alteration |
| W1__136815_2 | 100 | TaeA | Paenibacillus sp. LC231 | macrolide antibiotic;fluoroquinolone antibiotic;cephalosporin;penam;tetracycline antibiotic;peptide antibiotic;acridine dye;rifamycin antibiotic;pleuromutilin antibiotic;nitroimidazole antibiotic | antibiotic efflux |
| W1__33143_1 | 99.42 | AAC(6')-Ie-APH(2'')-Ia | Staphylococcus aureus | aminoglycoside antibiotic | antibiotic inactivation |
| W1__50496_2 | 100 | Clostridioides difficile gyrB conferring resistance to fluoroquinolone | Clostridium ljungdahlii DSM 13528 | fluoroquinolone antibiotic;aminocoumarin antibiotic | antibiotic target alteration |
| W1__7282_6 | 100 | Ureaplasma urealyticum gyrB conferring resistance to fluoroquinolone | Ureaplasma urealyticum serovar 10 str. ATCC 33699 | fluoroquinolone antibiotic;aminocoumarin antibiotic | antibiotic target alteration |
| W1__7282_7 | 100 | Staphylococcus aureus gyrA conferring resistance to fluoroquinolones | Staphylococcus aureus subsp. aureus MRSA252 | fluoroquinolone antibiotic;nybomycin | antibiotic target alteration |
| W1__73823_87 | 100 | Staphylococcus aureus gyrA conferring resistance to fluoroquinolones | Staphylococcus aureus subsp. aureus MRSA252 | fluoroquinolone antibiotic;nybomycin | antibiotic target alteration |
| W1__98663_3 | 80.3 | Clostridioides difficile gyrB conferring resistance to fluoroquinolone | Clostridium ljungdahlii DSM 13528 | fluoroquinolone antibiotic;aminocoumarin antibiotic | antibiotic target alteration |
| W2__118520_1 | 100 | cmeB | Campylobacter jejuni subsp. doylei 269.97 | macrolide antibiotic;fluoroquinolone antibiotic;monobactam;aminoglycoside antibiotic;carbapenem;cephalosporin;glycylcycline;penam;tetracycline antibiotic;acridine dye;aminocoumarin antibiotic;diaminopyrimidine antibiotic;phenicol antibiotic;fusidic acid;triclosan;antibacterial free fatty acids | antibiotic efflux |
| W2__140248_1 | 90.84 | Mycobacterium tuberculosis rpsA mutations conferring resistance to Pyrazinamide | Mycobacterium tuberculosis H37Rv | pyrazinamide | antibiotic target alteration |
| W2__141803_3 | 99.09 | Pseudomonas mutant PhoP conferring resistance to colistin | Pseudomonas aeruginosa PAO1 | macrolide antibiotic;fluoroquinolone antibiotic;cephalosporin;penam;tetracycline antibiotic;peptide antibiotic;acridine dye;rifamycin antibiotic;pleuromutilin antibiotic;nitroimidazole antibiotic | antibiotic target alteration;antibiotic efflux |
| W2__144059_3 | 74.41 | Staphylococcus aureus walK with mutation conferring daptomycin resistance | Staphylococcus aureus subsp. aureus MRSA252 | peptide antibiotic | antibiotic target alteration |
| W2__17770_3 | 100 | TaeA | Paenibacillus sp. LC231 | macrolide antibiotic;fluoroquinolone antibiotic;cephalosporin;penam;tetracycline antibiotic;peptide antibiotic;acridine dye;rifamycin antibiotic;pleuromutilin antibiotic;nitroimidazole antibiotic | antibiotic efflux |
| W2__33431_2 | 100 | Ureaplasma urealyticum gyrB conferring resistance to fluoroquinolone | Ureaplasma urealyticum serovar 10 str. ATCC 33699 | fluoroquinolone antibiotic;aminocoumarin antibiotic | antibiotic target alteration |
| W2__33431_3 | 100 | Staphylococcus aureus gyrA conferring resistance to fluoroquinolones | Staphylococcus aureus subsp. aureus MRSA252 | fluoroquinolone antibiotic;nybomycin | antibiotic target alteration |
| W2__49427_1 | 99.15 | AAC(6')-Ie-APH(2'')-Ia | Staphylococcus aureus | aminoglycoside antibiotic | antibiotic inactivation |
| W2__61754_1 | 99.51 | adeJ | Acinetobacter baumannii | macrolide antibiotic;fluoroquinolone antibiotic;monobactam;aminoglycoside antibiotic;lincosamide antibiotic;carbapenem;cephalosporin;glycylcycline;penam;tetracycline antibiotic;acridine dye;aminocoumarin antibiotic;rifamycin antibiotic;diaminopyrimidine antibiotic;phenicol antibiotic;triclosan;penem;antibacterial free fatty acids | antibiotic efflux |
| W2__77820_3 | 100 | Bartonella bacilliformis gyrB conferring resistance to aminocoumarin | Bartonella bacilliformis KC583 | aminocoumarin antibiotic | antibiotic target alteration |
| W2__78937_1 | 99.04 | mupB | Staphylococcus aureus | mupirocin | antibiotic target alteration |
| W2__96435_201 | 100 | Staphylococcus aureus gyrA conferring resistance to fluoroquinolones | Staphylococcus aureus subsp. aureus MRSA252 | fluoroquinolone antibiotic;nybomycin | antibiotic target alteration |
| W3__105078_3 | 92.97 | mupB | Staphylococcus aureus | mupirocin | antibiotic target alteration |
| W3__169162_2 | 99.65 | TEM-116 | Staphylococcus aureus | monobactam;cephalosporin;penam;penem | antibiotic inactivation |
| W3__22427_2 | 100 | Staphylococcus aureus parE conferring resistance to fluoroquinolones | Staphylococcus aureus subsp. aureus MRSA252 | fluoroquinolone antibiotic | antibiotic target alteration |
| W3__32997_97 | 100 | Staphylococcus aureus gyrA conferring resistance to fluoroquinolones | Staphylococcus aureus subsp. aureus MRSA252 | fluoroquinolone antibiotic;nybomycin | antibiotic target alteration |
| W3__8469_1 | 100 | TaeA | Paenibacillus sp. LC231 | macrolide antibiotic;fluoroquinolone antibiotic;cephalosporin;penam;tetracycline antibiotic;peptide antibiotic;acridine dye;rifamycin antibiotic;pleuromutilin antibiotic;nitroimidazole antibiotic | antibiotic efflux |
| W3__90179_2 | 98.9 | Clostridioides difficile gyrA conferring resistance to fluoroquinolones | Clostridioides difficile 630 | fluoroquinolone antibiotic;nybomycin | antibiotic target alteration |
| W3__90307_7 | 100 | Staphylococcus aureus gyrA conferring resistance to fluoroquinolones | Staphylococcus aureus subsp. aureus MRSA252 | fluoroquinolone antibiotic;nybomycin | antibiotic target alteration |
| W3__90307_8 | 100 | Ureaplasma urealyticum gyrB conferring resistance to fluoroquinolone | Ureaplasma urealyticum serovar 10 str. ATCC 33699 | fluoroquinolone antibiotic;aminocoumarin antibiotic | antibiotic target alteration |
